# Supplementary material for: Barriers to Accessing Maternal Care in Low Income Countries in Africa: A Systematic Review
Source: Int J Environ Res Public Health. 2020 Jun 16;17(12):4292. doi: 10.3390/ijerph17124292 (PMC7344902; doi:10.3390/ijerph17124292)
Supplement: Supplementary file 1 [file ijerph-17-04292-s001.pdf]

Table S1: Search Strategy

Search Number	Search Term
1	maternal care Keyword
2	(pregnancy or maternal or obstetric or perinatal or antenatal) keywords
3	maternal services keywords
4	pregnancy/ or exp labor, obstetric/ or exp pregnancy outcome/ or exp pregnancy, high-risk/ (subject headings; applicable for Medline, Global Health and CINAHL Plus)
5	exp Maternal Health/ or exp Maternal Behavior/ or exp Maternal Health Services/ (subject headings; applicable for Medline, Global Health and CINAHL Plus)
6	((matern* or ANC) adj (care or health) Keywords with proximity
7	(matern* adj facilit*) keywords allowing for truncation
8	1 or 2 or 3 or 4 or 5 or 6 or 7 **(all relevant to concept A)**
9	(barrier* or access or difficult* or challenge* or obstacle* or problem* or socio* or transport* or stigma or cultur* or equit*) keywords
10	exp "Patient Acceptance of Health Care"/ or exp Maternal Health Services/ or exp Health Services Accessibility/ (subject headings; applicable for Medline, Global Health and CINAHL Plus)
11	9 or 10 **(concept B)**
12	africa, central/ or exp central african republic/ or exp chad/ or exp congo/ or exp "democratic republic of the congo"/ or africa, eastern/ or burundi/ or exp eritrea/ or exp ethiopia/ or kenya/ or exp rwanda/ or exp somalia/ or exp south sudan/ or sudan/ or exp tanzania/ or exp uganda/ or exp malawi/ or exp mozambique/ or exp zimbabwe/ or exp benin/or exp burkina faso/ or exp gambia/ or exp guinea/ or exp guinea-bissau/ or exp liberia/ or exp mali/ or exp niger/ or senegal/ or exp sierra leone/ or exp togo/ (subject headings; applicable for Medline, Global Health and CINAHL Plus)
13	Africa/ (subject headings; applicable for Medline, Global Health and CINAHL Plus)

14	(Benin or Burkina Faso or Burundi or Central African Republic or Chad or Comoros or Congo or Eritrea or Ethiopia or Gambia or Guinea or Guinea Bissau or Liberia or Madagascar or Malawi or Mali or Mozambique or Niger or Rwanda or Senegal or Sierra Leone or Somalia or South Sudan or Tanzania or Togo or Uganda or Zimbabwe) keywords
15	12 or 13 or 14 (subject headings; applicable for Medline, Global Health and CINAHL Plus) **(Concept C)**
16	8 and 11 and 15<br>Concept A **AND** Concept B **AND** Concept C

Table S2: Search Strategy for each database

Search strategy on Medline: (18 May 2019)

**Search Number**	**Search Term**	**# results in Medline**
1	maternal care.mp. [mp=title, abstract, original title, name of substance word, subject heading word, floating sub-heading word, keyword heading word, organism supplementary concept word, protocol supplementary concept word, rare disease supplementary concept word, unique identifier, synonyms]	**(2108)**
2	(pregnancy or maternal or obstetric or perinatal or antenatal).mp. [mp=title, abstract, original title, name of substance word, subject heading word, floating sub-heading word, keyword heading word, organism supplementary concept word, protocol supplementary concept word, rare disease supplementary concept word, unique identifier, synonyms]	**(998361)**
3	maternal services.mp. [mp=title, abstract, original title, name of substance word, subject heading word, floating sub-heading word, keyword heading word, organism supplementary concept word, protocol supplementary concept word, rare disease supplementary concept word, unique identifier, synonyms]	**(75)**
4	exp Maternal Health/ or exp Maternal Behavior/ or exp Maternal Health Services/	**(57786)**
5	((matern* or ANC) adj (care or health)).mp. [mp=title, abstract, original title, name of substance word, subject	**(21461)**

	heading word, floating sub-heading word, keyword heading word, organism supplementary concept word, protocol supplementary concept word, rare disease supplementary concept word, unique identifier, synonyms]	
6	(matern* adj facilit*).mp. [mp=title, abstract, original title, name of substance word, subject heading word, floating sub-heading word, keyword heading word, organism supplementary concept word, protocol supplementary concept word, rare disease supplementary concept word, unique identifier, synonyms]	**(113)**
7	1 or 2 or 3 or 4 or 5 or 6	**(1000524)**
8	(barrier* or access or difficult* or challenge* or obstacle* or problem* or socio* or transport* or stigma or cultur* or equit*).mp. [mp=title, abstract, original title, name of substance word, subject heading word, floating sub-heading word, keyword heading word, organism supplementary concept word, protocol supplementary concept word, rare disease supplementary concept word, unique identifier, synonyms]	**(4235633)**
9	exp "Patient Acceptance of Health Care"/ or exp Maternal Health Services/ or exp Health Services Accessibility/	**(278889)**
10	8 or 9	**(4407589)**
11	africa, central/ or exp central african republic/ or exp chad/ or exp congo/ or exp "democratic republic of the congo"/ or africa, eastern/ or burundi/ or exp eritrea/ or exp ethiopia/ or exp rwanda/ or exp somalia/ or exp south sudan/ or sudan/ or exp tanzania/ or exp uganda/ or exp malawi/ or exp mozambique/ or exp zimbabwe/ or exp benin/ or exp burkina faso/ or exp gambia/ or exp guinea/ or exp guinea-bissau/ or exp liberia/ or exp mali/ or exp niger/ or senegal/ or exp sierra leone/ or exp togo/ or exp comoros/ or exp madagascar/	**(85096)**
12	Africa/	**(28602)**
13	(Benin or Burkina Faso or Burundi or Central African Republic or Chad or Comoros or Congo or Eritrea or Ethiopia or Gambia or Guinea or Guinea Bissau or Liberia or Madagascar or Malawi or Mali or Mozambique or Niger or Rwanda or	**(261596)**

	Senegal or Sierra Leone or Somalia or South Sudan or Tanzania or Togo or Uganda or Zimbabwe).mp. [mp=title, abstract, original title, name of substance word, subject heading word, floating sub-heading word, keyword heading word, organism supplementary concept word, protocol supplementary concept word, rare disease supplementary concept word, unique identifier, synonyms]	
14	11 or 12 or 13	**(292381)**
15	7 and 10 and 14	**(8549)**
16	limit 15 to (english language and yr="2015 -Current")	**(2445)**

Search Strategy on Global Health: (18 May 2019)

**Search Number**	**Search Term**	**#results in Global Health**
1	maternal care.mp. [mp=abstract, title, original title, broad terms, heading words, identifiers, cabicodes]	**(627)**
2	(pregnancy or maternal or obstetric or perinatal or antenatal).mp. [mp=abstract, title, original title, broad terms, heading words, identifiers, cabicodes]	**(149438)**
3	maternal services.mp. [mp=abstract, title, original title, broad terms, heading words, identifiers, cabicodes]	**(70)**
4	exp Maternal Health/ or exp Maternal Behavior/ or exp Maternal Health Services/	**(0)**
5	((matern* or ANC) adj (care or health)).mp. [mp=abstract, title, original title, broad terms, heading words, identifiers, cabicodes]	**(4488)**

6	(matern* adj facilit*).mp. [mp=abstract, title, original title, broad terms, heading words, identifiers, cabicodes]	**(72)**
7	1 or 2 or 3 or 4 or 5 or 6	**(149580)**
8	(barrier* or access or difficult* or challenge* or obstacle* or problem* or socio* or transport* or stigma or cultur* or equit*).mp. [mp=abstract, title, original title, broad terms, heading words, identifiers, cabicodes]	**(806973)**
9	exp "Patient Acceptance of Health Care"/ or exp Maternal Health Services/ or exp Health Services Accessibility/	**(0)**
10	8 or 9	**(806973)**
11	africa, central/ or exp central african republic/ or exp chad/ or exp congo/ or exp "democratic republic of the congo"/ or africa, eastern/ or burundi/ or exp eritrea/ or exp ethiopia/ or exp rwanda/ or exp somalia/ or exp south sudan/ or sudan/ or exp tanzania/ or exp uganda/ or exp malawi/ or exp mozambique/ or exp zimbabwe/ or exp benin/ or exp burkina faso/ or exp gambia/ or exp guinea/ or exp guinea-bissau/ or exp liberia/ or exp mali/ or exp niger/ or senegal/ or exp sierra leone/ or exp togo/ or exp comoros/ or exp madagascar/	**(93368)**
12	Africa/	**(247594)**
13	(Benin or Burkina Faso or Burundi or Central African Republic or Chad or Comoros or Congo or Eritrea or Ethiopia or Gambia or Guinea or Guinea Bissau or Liberia or Madagascar or Malawi or Mali or Mozambique or Niger or Rwanda or Senegal or Sierra Leone or Somalia or South Sudan or Tanzania or Togo or Uganda or Zimbabwe).mp. [mp=abstract, title, original title, broad terms, heading words, identifiers, cabicodes]	**(145142)**
14	11 or 12 or 13	**(298382)**
15	7 and 10 and 14	**(9015)**
16	limit 15 to (english language and yr="2015 -Current")	**(3315)**

Search Strategy for CINAHL Plus: (Applied on 18th May 2019)

Search Number	Search Term	#results in CINAHL Plus
1	MH "Maternal-Child Health") OR (MH "Maternal Health Services+")OR (MH "Health Services Needs and Demand+") OR (MH "Attitude of Health Personnel+") OR (MH "Personnel, Health Facility+") OR (MH "Maternal Behavior") OR (MH "Health Resource Utilization/UT/ED/EC/MA/MT/TD") OR (MH "Health Personnel+")	**(591,255)**
2	maternal care	**(552)**
3	(MH "Labor+")	**(11,712)**
4	maternal service	**(44)**
5	pregnancy or maternal or obstetric or perinatal or antenatal	**(257,472)**
6	(MH "Delivery, Obstetric+") OR (MH "Obstetric Care+")	**(43,256)**
7	ANC	**(1,004)**
8	S1 OR S2 OR S3 OR S4 OR S5 OR S6 OR S7	**(808,807)**
9	barrier or access or difficulty or challenge or obstacle or problem or socioeconomic or transport or stigma or culture or equity	**(752,672)**
10	health services accessibility	**(73,052)**
11	(MH "Health Services Accessibility+") OR (MH "Health Services Needs and Demand+") OR (MH "Maternal Health Services+")	**(114,396)**
12	S9 OR S10 OR S11	**(820,391)**
13	Benin or Burkina Faso or Burundi or Central African Republic or Chad or Comoros or Congo or Eritrea or Ethiopia or Gambia or Guinea or Guinea Bissau or Liberia or Madagascar or Malawi or Mali or Mozambique or Niger or Rwanda or Senegal or Sierra Leone or Somalia or South Sudan or Tanzania or Togo or Uganda or Zimbabwe	**(29,302)**
14	(MH "Chad") OR (MH "Congo") OR (MH "Burundi") OR (MH "Eritrea") OR (MH "Ethiopia") OR (MH "Rwanda") OR (MH "Somalia") OR (MH "Sudan") OR (MH "Tanzania") OR (MH "Uganda") OR (MH "Malawi") OR (MH "Mozambique") OR (MH "Zimbabwe") OR (MH "Benin") OR (MH "Burkina Faso") OR (MH	**(20,770)**

	"Gambia") OR (MH "Guinea") OR (MH "Guinea-Bissau") OR (MH "Liberia") OR (MH "Mali") OR (MH "Niger") OR (MH "Sierra Leone")<br>OR (MH "Togo") OR (MH "Central African Republic") OR (MH "Comoros") OR (MH "Madagascar") OR (MH "Senegal")	
15	(MH "Africa")	**(7,408)**
16	S13 OR S14 OR S15	**(36,838)**
17	S8 AND S12 AND S16	**(4,393)**
18	S8 AND S12 AND S16 Limiters - Published<br>Date: 20150101-20190531; English Language<br>Search modes -Boolean/Phrase	**(2,180)**

Table S3: MMAT Appraisal Results

MMAT for Qualitative Research

**Author/ Year (Study)**	**Are there clear research questions?**	**Do the collected data allow to address the research questions?**	**1.1. Is the qualitative approach appropriate to answer the research question?**	**1.2. Are the qualitative data collection methods adequate to address the research question?**	**1.3. Are the findings adequately derived from the data?**	**1.4. Is the interpretation of results sufficiently substantiated by data?**	**1.5. Is there coherence between qualitative data sources, collection, analysis and interpretation?**	**Score**
D Musoke et al. 2015	Yes	Yes	Yes	Can't tell	Can't tell	Can't tell	Yes	40 %
S. MacVica	Yes	Yes	Can't tell	Yes	Yes	Yes	Can't tell	60 %

r et al. 2017								
Kaba et al., 2017 (32)	Yes	Yes	Yes	Yes	Yes	Yes	Yes	100 %
Nyathi et al., 2017 (34)	Yes	Yes	Yes	Yes	Yes	Yes	Yes	100 %
Wilunda et al., 2017 (35)	Yes	Yes	Yes	Yes	Yes	Yes	Yes	100 %
Madula et al., 2018 (36)	Yes	Yes	Yes	Yes	Yes	Yes	Yes	100 %
Colley et al. 2018	Yes	Can't tell						0%
Kohi et al., 2018 (38)	Yes	Yes	Yes	Yes	Yes	Yes	Yes	100 %
Mugo et al., 2018 (39)	Yes	Yes	Yes	Yes	Yes	Yes	Yes	100 %
Ahmed et al., 2018 (40)	Yes	Yes	Yes	Yes	Yes	Yes	Yes	100 %
Wilunda et al., 2016 (28)	Yes	Yes	Yes	Yes	Yes	Yes	Yes	100 %
Arnold et al., 2016 (29)	Yes	Yes	Yes	Can't tell	Yes	Yes	Yes	80 %
Munyaradzi Kenneth et al. 2016	Can't tell	Can't tell						0%

Munguambe et al., 2016 (30)	Yes	Yes	Yes	Yes	Yes	Yes	Yes	100 %
Burrowes et al., 2017 (31)	Yes	Yes	Yes	Yes	Yes	Yes	Yes	100 %
Medhanyie et al., 2018 (37)	Yes	Yes	Yes	No	Yes	Yes	Yes	80 %

MMAT for Mixed Method Studies

**Author/ Year (Study)**	**Are there clear research questions?**	**Do the collected data allow to address the research questions?**	**5.1. Is there an adequate rationale for using a mixed methods design to address the research question?**	**5.2. Are the different components of the study effectively integrated to answer the research question?**	**5.3. Are the outputs of the integration of qualitative and quantitative components adequately interpreted?**	**5.4. Are divergences and inconsistencies between quantitative and qualitative results adequately addressed?**	**5.5. Do the different components of the study adhere to the quality criteria of each tradition of the methods involved?**	**Score**
N. Alam et al. 2016	Yes	Yes	Yes	Yes	Yes	Can't tell	Can't tell	60%
Mamba et al. 2017	Yes	Yes	Yes	Yes	Yes	No	No	60%

Lawry et al., 2017 (33)	Yes	Yes	Yes	Yes	Yes	Yes	Yes	100 %
Mhlanga et al. 2017	Yes	Yes	Yes	Yes	Can't tell	No	Can't tell	60%
Andarge et al. 2017	Yes	Yes	Yes	Can't tell	Can't tell	Yes	Yes	60%

MMAT assessment for each study

**Author/Year (Study)**	**Comments**
D Musoke et al. 2015	1- not very accurate results driven from Photovoice. Some photographs were interpreted by the researcher and not based on the real experience of the participant.<br>2- Results of photovoice could be subjective; based on participant's personal perception or experience. This should be considered by researchers while interpreting results
N. Alam et al. 2016	1- Use of structured interviews might have missed out on other variables and factors that are not in the prepared list of interview questions.<br>2- Having semi-structured interviews with more room for participants to share their experience were used to explore broader themes to cover this area (strength)<br>3- Inconsistency in outputs and limitations of qualitative and quantitative work was not fully explained, it was only mentioned that the purposive sampling didn't allow for generalizability of results. Moreover, the combination of qualitative and quantitative methods allowed for triangulation
S. MacVicar et al. 2017	The study relied on FDGs and interviews with only 16 pregnant women, and 10 stakeholders; the results of the study cannot be generalized.<br>The aim of the study was not only to identify barriers to access maternal care with respect to weather and seasonality. It was only hinted out in the results section, but without mentioning how many participants actually reported this as one of the main challenges.
Mamba et al. 2017	1- English is an official language in Malawi, which makes it more accurate to interpret the results and answers to the interviews, with less potential of mis-interpretation

	2- Mixed method approach allowed for triangulation to further provide significance to the results (combining both qualitative and quantitative approaches).<br>3- Sample size was huge (positive for data analysis)<br>4- The study was part of a community program already established, so there is potential bias in sampling and in interpreting the data. It would have been better to perform the study independent of the Community based program, or by independent researchers. Sample is not representative of the total population.<br>5- Translators were 2 secondary school teachers; would have been more reliable to include 2 different translators with 2 different backgrounds, or more certified to ensure quality of translation<br>6- Participants were of both groups; those attending early and late to ANC visits<br>7- Quantitative method was only measured as frequencies (Poor analysis)
Kaba et al., 2017 (32)	1- Using explorative qualitative method is convenient to investigate reasons for non-use of maternal health services by women in urban slums; a topic that has not been studied in depth before.<br>2- Sampling from districts with known vulnerability--> slum urban, with poor socio-economic people living there<br>3- Data collection was based on choosing areas where it is assumed to have less utilization of maternal health services (Weak, it should be based on evidence)<br>4- There was no pre-testing for the interviews prior to study conduction to ensure their validity.<br>5- The researcher clarified how the data collection (in-depth interviews and FGDs) and analysis will be done, and he used 3 independent researchers for interview transcription; to increase quality of reported themes and sub-themes into the thematic analysis of the results.<br>6- The researcher has not discussed saturation of data<br>7- The researcher has not identified or clarified potential bias and study limitations. This reduces the strength of results for this study. There could be potential selection and responder bias. FGDs for vulnerable women could have influenced their responses. They could have been more conservative or afraid of disclosing all the reasons
Lawry et al., 2017 (33)	1- Limitation of not being able to access some villages due to rainy seasons and roads hard to walk in, missed out on reaching these areas and their potentially important barriers to access healthcare. This hinders generalizability of the results of the study. But this was hinted to in the limitations (+ve)

	2- The qualitative method relied on open ended questions which gave the opportunity to reveal all possible barriers to be reported by participants (women, men, key informants, stakeholders, community leaders)
Mhlanga et al. 2017	1- Study limitation not mentioned, and quality of qualitative method was not explained or clarified in the article.<br>2- The study reports briefly on access and utilization barriers to maternal care, with no elaborated quantitative and qualitative analysis on this topic

Nyathi et al., 2017 (34)	1- Using explorative qualitative method is convenient to investigate the different factors for accessibility and utilization of ANC services<br>2- Explanation for the chosen settings was not very clear in the researcher's description of the methodology<br>3- Semi-structured interviews were used and pre-tested for validity.<br>4- Lack of explanation of the selection criteria introduces potential selection and response bias, as there was no explanation of the nature of culture in this district, the socio-economic level of the population living there, their educational level, and proximity to health facilities.<br>5- Duration of interviews conducted was appropriate<br>6- The researcher did not acknowledge weakness and limitations, potential selection bias as they chose only poor districts which is assumed to have difficulties in access to healthcare in general, generalizability cannot be implied from the results<br>7- Although the researcher adapted the Lincoln and Guba model to address dependability, credibility, transferability and confirmability of the study results, he didn't acknowledge his study limitations and potential biases.<br>8- No generalizability and triangulation of results
Andarge et al. 2017	Study mainly discussing prevalence and factors associated with birth preparedness, with minimal focus on what hinders women from access to maternal facilities and ANC visits. There was no clear integration of both qualitative and quantitative results and why they were used to enhance the results or provide more rigorous outputs
Wilunda et al., 2017 (35)	1- The research design, choice of the setting was adequate and in accordance with the research question and the selection criteria for the required participants.<br>2- Sound choice of study settings and selection criteria<br>3- The researcher explained that he will perform KIIs and FGDs for stakeholders and pregnant women respectively. The researcher has done the pre-testing for the questions prior to conducting the interviews to ensure the validity of the questions. On the other

	hand, only 20 minutes was the time given for the interviews, which could be a short time and not giving participants enough time and space to respond fully and probe on what they perceive as potential barriers.<br>4- Having some transcripts for interviews missed, but this was addressed in the discussion of the paper (+ve)<br>5- Although study limitations were acknowledged, the research lacked mentioning potential biases
Madula et al., 2018 (36)	1- Qualitative method is suitable for describing the nature of communication between health providers and women in maternal health services in Malawi<br>2- The researcher decided on addressing this aim as he referred to evidence on the poor health providers' attitude and behavior with patients in Malawi that urged to investigate this issue through the researcher's work<br>3- The researcher showed good recruitment strategy. Systematic random sampling was adapted. The researcher decided to include participants from all 3 regions of Malawi; from public and private hospitals, to ensure variability and diversity of the chosen sample. This gives a chance for generalizable results for Malawi as a country; and not a specific region. The decision of selection criteria was wise; women who gave birth in the last 5 days; this reduces any chances of recall bias.<br>4- The researcher adequately justified his data collection settings and recruitment strategy.<br>5- The researcher explained that semi-structured interviews will be held for participants with follow up questions to probe and provide more in-depth replies.<br>6- The researcher clarified that he used an interview guide adapted from previous literature and results were recorded and transcribed.<br>7- The researcher has indicated data saturation and that he continued even beyond saturation point to allow for heterogeneity.<br>8- The researcher mentioned that he will participate in conducting the interviews and data analysis, this could increase potential researcher bias as the researcher could be influenced by his own hypothesis and perception and could have indirectly guided the responses of the participants.<br>9- The researcher failed to address the potential bias of the results, even though he was aware of some other potential limitations as mentioned in his discussion<br>10- No triangulation of results
Colley et al. 2018	Exclude this article as the aim of the research was not well identified through the results, also it had no clear correlation to dignity and respect during

	childbirth, which is one of the factors that might affect access of women to maternal care
Kohi et al., 2018 (38)	1- Qualitative methods seem appropriate for the exploratory objectives of the study.<br>2- Convenience sampling based on the eligibility criteria. However, there was no clear explanation for the researcher's recruitment selection criteria.<br>3- The researcher has chosen an appropriate setting and provided a convincing justification as this area had one of the highest maternal mortality rates<br>4- Clear description for data collection; semi-structured interviews and FGDs using interview guide.<br>5- Data saturation was taken into consideration and discussed in the methodology section<br>6- Limitations were mentioned in the article, it only reported results for women and men who used health facilities, so the results are not generalizable and not capturing barriers to other women not accessing HF, biased results. (These limitations and biases were acknowledged by the researcher in the article)
Mugo et al., 2018 (39)	1- Purposive sampling used to select areas with accessibility and relative security; which is not generalizable to the total population, however this was pointed at in the limitations section at the end of the article.<br>2- Study settings were well explained<br>3- The researcher explained the data collection and clarified that he will be using in-depth one to one interviews with women and their husbands to explore their perceived barriers to access maternal healthcare. Data saturation was not discussed in the study in addition to the duration of each interview.<br>4- During translating the interview responses, there could be some missed information or misinterpretations.<br>5- The researcher highlighted that he adapted a conceptual framework and modified its themes according to the research question. Themes were adapted according to the study results in accordance with the research aim.<br>6- The researcher failed to address potential bias
Ahmed et al., 2018 (40)	1- The researcher has adequately justified his aim for this research due to the limited available data on determinants of assisted childbirth by nomadic women.<br>2- The researcher has explained the reason for study settings (Gossi in Mali), as it contains 90% nomadic people

	3- Purposive and convenience sampling due to limited fund available for the research and for security reasons due to the conflicting situation in Mali.<br>4- The researcher has tried to include a diversity of the population for generalizability<br>5- The researcher took into consideration selecting women who gave birth 3 months preceding data collection in order to reduce potential recall bias<br>6- Pre-testing of interviews was considered and done to ensure validity. Moreover, the choice of conducting one to one interviews was to reduce potential response bias.<br>7- The researcher has considered data saturation in his methodology<br>8- The researcher successfully accounted for recall and response bias in his study and addressed study limitations<br>9- The researcher accounted for validity and reliability of his results.
Wilunda et al., 2016 (28)	1- Research aim was well justified and explained<br>2- Qualitative method was the best approach for this study aim, Using FDGs and KIIs allow exploration of the variable barriers and allow triangulation of resulted reasons reported by pregnant women and health providers.<br>3- The researcher fully explained his methodology which seems appropriate for addressing his aim<br>4- Random selection for women and their husbands for FDGs was suitable in an attempt to generalize the results. The researcher randomly selected participants from different areas and backgrounds.<br>5- KIIs however to interview HCWs was purposive, which might have increased risk of reporting bias. However, the researcher explained that the reason for this purposive sampling was to interview health professionals specialized in maternal health; which is valid.<br>6- Data collection methods were adequately explained and justified. The researcher paid due attention to the privacy of FDGs and KIIs, with cultural and language considerations. Ethical considerations were respected for this study for all participants and informed consents were obtained prior to FDGs or KIIs.
Arnold et al., 2016 (29)	1- Clear aim<br>2- Semi-structured interviews were used as a suitable tool to explore stakeholders' perspective, while FDGs were used as an adequate method to approach women and identify their perceived barriers to access maternal health.<br>3- The researcher fully explained his methodology which seems appropriate for addressing his aim<br>4- Good selection criteria were chosen with justification. Even though purposive sampling could lead to biased results, but it was convenient

	and addressing the research question for investigating a region in Togo. However, the results could not be generalized.<br>5- The setting for data collection was suitable to the location targeted in the research aim. There was a clear description that there will be KIIs and FGDs that are guided by previous semi-structured interviews. Pre-testing of the methodological tools prior to data collection was a strength. Data collection and interpretation were squeezed in a very short period of time, that could reduce the quality of the produced transcripts.<br>6- Results were not generalizable and cannot be transferred to other populations. Another thing is that the researcher mentioned at the beginning of the article that gender inequality contributed to access barriers to reproductive and maternal health. However, the results did not fully reflect on that; it was only reported as one of the identified themes of the study.
Munyaradzi Kenneth et al. 2016	Low quality data and lack of clear research question
Munguambe et al., 2016 (30)	1- Using qualitative methodology to explore ethnographic aspects of accessing maternal health is the most optimum method to investigate health seeking behavior that could influence the utilization of maternal health.<br>2- KIIs and FGDs are suitable for interviewing HCWs and women respectively, to identify their perceived barriers to access maternal health. Understanding the perspectives of both parties allowed for triangulation of results.<br>3- The researcher fully explained his methodology which seems appropriate for addressing his aim<br>4- Purposively selection to ensure diversity of socio-economic status, cultural and structural factors that might contribute to variability in utilization and access of maternal health services. The selection criteria were explained for decision-makers, community members and HCWs included in this study.<br>5- Clear setting was identified and explained for this study, which fits the research aim. KIIs and FGDs were used as suitable methods to explore the health seeking behavior for maternal health services. The researcher has done pre-testing and quality check for the interviews performed. Thorough caution was given to ensure total confidentiality and comfort during the interviews. Same-gender interviewers were selected for participants to provide a more relaxed, comfortable environment to facilitate exploring all gender sensitive issues.<br>6- For some parts of methodology, the researcher clearly explained his role such as the decision of same-gender interviewers to ensure

	comfortable and confidential atmosphere during one to one interviews. Moreover, the researcher has taken into consideration the setting selected for recruitment to guarantee diversity and relative generalizable results.<br>7- No reporting of any potential biases and how they shall be addressed. In addition, the study limitations were not thoroughly explained in this study.
Burrowes et al., 2017 (31)	1- Qualitative cross-sectional methods are suitable to explore the disrespect and abuse during childbirth. Using open-ended interviews for women who recently delivered, and in-depth interviews for midwives is adequate for addressing the research aim.<br>2- Pre-testing for questionnaires and methodology tools was done prior to conducting the study. The study does not allow for generalizability, and the sample size was small and could not stratify the results based on different possible confounders (socio-economic status of patients and their education)<br>3- Using convenience and purposive sampling for recruitment could increase the chances of selection bias. Moreover, the sample recruited will not be representing the whole population and consequently the study results can't be generalized<br>4- The researcher explained the background of the interviewers, and how they were selected to reduce researcher bias. Moreover, the researcher clarified how the data will be collected , transcribed, coded and analyzed. He even mentioned some of the challenges in the coding, which strengthens the study, as the researcher is aware of his potential weaknesses and how to address them.<br>5- Verbal consent for women was obtained, and the study was only effective after receiving ethical committee approval from the Debre Markos University<br>6- The researcher highlighted the discrepancies in results between women and midwives and tried to justify them based on the recruitment and sampling strategies used. The researcher had also solid awareness of the study limitations and pointed them out clearly in the discussion section.
Medhanyie et al., 2018 (37)	1- Using qualitative methods is appropriate to explore the variable barriers to utilize reproductive and maternal health services among the pastoralist communities in Ethiopia.<br>2- The purposive selection of pastoralist communities was justified by their unique characteristics, culture and socio-economic statuses that might have influenced their behavior and utilization of reproductive and maternal health

	3- The selected population was drawn from all 5 regions of Afar, to ensure diversity and full coverage of the pastoralist community<br>4- The selected population was drawn from all 5 regions of Afar, to ensure diversity and full coverage of the pastoralist community and data validity was considered<br>5- Data analysis was properly done but the researcher failed to address the study limitations and potential biases
